# (*E*)-*N*′-(3,4-Dimethoxy­benzyl­idene)-2,4-dihydroxy­benzohydrazide methanol solvate

**DOI:** 10.1107/S1600536809035752

**Published:** 2009-09-09

**Authors:** Qiao-Ling Zhang, Li-Zi Yin, Xu-Ming Deng, Song-Cai Liu, De-Guang Song

**Affiliations:** a College of Animal Science and Veterinary Medicine, Jilin University, Changchun 130062, People’s Republic of China

## Abstract

The title compound, C_16_H_16_N_2_O_5_·CH_3_OH, was obtained from a condensation reaction of 3,4-dimethoxy­benzaldehyde and 2,4-dihydroxy­benzohydrazide. The non-H atoms of the Schiff base mol­ecule are approximately coplanar (r.m.s. deviation = 0.043 Å) and the dihedral angle between the two benzene rings is 1.6 (1)°. The mol­ecule adopts an *E* configuration with respect to the C=N double bond. An intra­molecular O—H⋯O hydrogen bond is observed. The Schiff base and methanol mol­ecules are linked into a two-dimensional network parallel to (10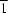) by inter­molecular N—H⋯O, O—H⋯N and O—H⋯O hydrogen bonds.

## Related literature

For background to Schiff base compounds, hydrazone compounds and their biological properties, see: Kucukguzel *et al.* (2006 ▶); Khattab *et al.* (2005 ▶); Karthikeyan *et al.* (2006 ▶); Okabe *et al.* (1993 ▶). For bond-length data, see: Allen *et al.* (1987 ▶). For related structures, see: Shan *et al.* (2008 ▶); Fun *et al.* (2008 ▶); Ma *et al.* (2008 ▶); Diao *et al.* (2008*a* ▶,*b* ▶); Ejsmont *et al.* (2008 ▶).
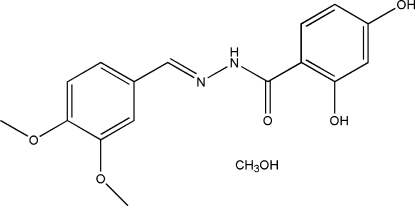


## Experimental

### 

#### Crystal data

C_16_H_16_N_2_O_5_·CH_4_O
*M* *_r_* = 348.35
Monoclinic, 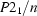

*a* = 8.497 (1) Å
*b* = 17.431 (2) Å
*c* = 11.933 (2) Å
β = 102.93 (2)°
*V* = 1722.6 (4) Å^3^
*Z* = 4
Mo *K*α radiation
μ = 0.10 mm^−1^
*T* = 298 K
0.25 × 0.23 × 0.23 mm

#### Data collection

Bruker SMART CCD area-detector diffractometer
Absorption correction: multi-scan (*SADABS*; Bruker, 2001 ▶) *T*_min_ = 0.975, *T*_max_ = 0.977
10465 measured reflections
3732 independent reflections
2017 reflections with *I* > 2σ(*I*)
*R*_int_ = 0.043

#### Refinement

*R*[*F* ^2^ > 2σ(*F* ^2^)] = 0.050
*wR*(*F* ^2^) = 0.129
*S* = 1.03
3732 reflections
235 parameters
1 restraint
H atoms treated by a mixture of independent and constrained refinement
Δρ_max_ = 0.15 e Å^−3^
Δρ_min_ = −0.19 e Å^−3^

### 

Data collection: *SMART* (Bruker, 2007 ▶); cell refinement: *SAINT* (Bruker, 2007 ▶); data reduction: *SAINT*; program(s) used to solve structure: *SHELXTL* (Sheldrick, 2008 ▶); program(s) used to refine structure: *SHELXTL*; molecular graphics: *SHELXTL*; software used to prepare material for publication: *SHELXTL*.

## Supplementary Material

Crystal structure: contains datablocks global, I. DOI: 10.1107/S1600536809035752/ci2900sup1.cif

Structure factors: contains datablocks I. DOI: 10.1107/S1600536809035752/ci2900Isup2.hkl

Additional supplementary materials:  crystallographic information; 3D view; checkCIF report

## Figures and Tables

**Table 1:** Hydrogen-bond geometry (Å, °)

*D*—H⋯*A*	*D*—H	H⋯*A*	*D*⋯*A*	*D*—H⋯*A*
N1—H1*A*⋯O2^i^	0.90 (1)	2.217 (10)	3.108 (2)	170 (2)
O6—H6⋯N2^ii^	0.82	2.55	3.133 (2)	129
O6—H6⋯O3^ii^	0.82	2.02	2.807 (2)	161
O2—H2⋯O6^iii^	0.82	1.79	2.599 (2)	171
O1—H1⋯O3	0.82	1.80	2.534 (2)	148

Symmetry codes: (i) 
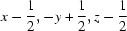
; (ii) 
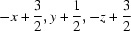
; (iii) 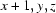.

**Fig. 1.**
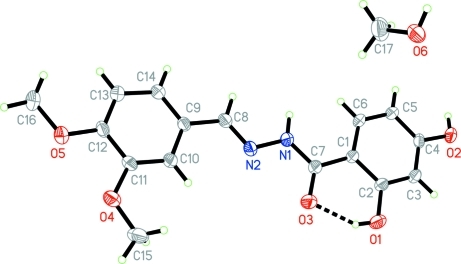
The asymmetric unit of the title compound. Displacement ellipsoids are drawn at the 30% probability level. The dashed line indicates an intramolecular hydrogen bond.

**Fig. 2.**
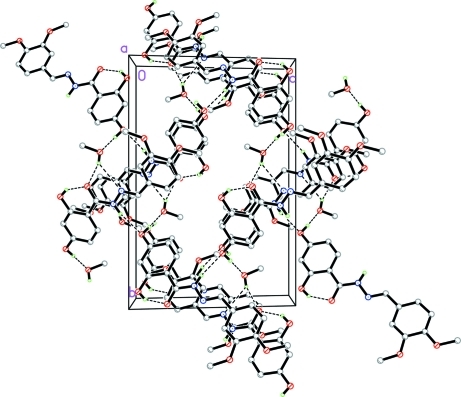
The molecular packing of the title compound, viewed along the a axis. H atoms not involved in hydrogen bonding (dashed lines) have been omitted for clarity.

## supplementary crystallographic information

### Comment

Hydrazones and Schiff bases have been attracted much attention for their excellent biological properties, especially for their potential pharmacological and antitumor properties (Kucukguzel *et al.*, 2006; Khattab *et al.*, 2005; Karthikeyan *et al.*, 2006; Okabe *et al.*, 1993). Recently, a large number of hydrazone derivatives have been prepared and structurally characterized (Shan *et al.*, 2008; Fun *et al.*, 2008; Ma *et al.*, 2008; Diao *et al.*, 2008*a*,b; Ejsmont *et al.*, 2008). As part of the ongoing study, we report herein the crystal structure of the title compound.

The molecular structure of the title compound is shown in Fig. 1. The compound consists of a Schiff base molecule and a methanol molecule. The dihedral angle between the two benzene rings is 1.6 (1)°. The Schiff base molecule displays an *E* configuration about the C═N bond. The bond lengths are typical (Allen *et al.*, 1987). The molecules are linked into a two-dimensional network  parallel to the (101) by intermolecular N—H···O, O—H···N, and O—H···O hydrogen bonds (Fig. 2 and Table 1).

### Experimental

3,4-Dimethoxybenzaldehyde (1.0 mmol, 166.2 mg) was dissolved in methanol (50 ml), then 2,4-dihydroxybenzohydrazide (1.0 mmol, 168.2 mg) was added slowly into the solution, and the mixture was kept at reflux with continuous stirring for 1 h. After the solution had cooled to room temperature colourless crystallites appeared. The crystallites were filtered and washed with methanol for three times. Recrystallization from an absolute methanol yielded block-shaped single crystals of the title compound.

### Refinement

Atom H1A was located from a difference Fourier map and refined isotropically, with the N-H distance restrained to 0.90 (1) Å. Other H atoms were placed in calculated positions with C-H = 0.93-0.96 Å and O-H = 0.82 Å, and refined in riding mode with *U*_iso_(H) = 1.2*U*_eq_(C) and 1.5*U*_eq_(O and C_methyl_). A rotating group model was used for methyl and hydroxyl groups.

### Crystal data

**Table:**

C_16_H_16_N_2_O_5_·CH_4_O	*F*(000) = 736
*M**_r_* = 348.35	*D*_x_ = 1.343 Mg m^−^^3^
Monoclinic, *P*2_1_/*n*	Mo *K*α radiation, λ = 0.71073 Å
Hall symbol: -P 2yn	Cell parameters from 1327 reflections
*a* = 8.497 (1) Å	θ = 2.3–24.5°
*b* = 17.431 (2) Å	µ = 0.10 mm^−^^1^
*c* = 11.933 (2) Å	*T* = 298 K
β = 102.93 (2)°	Block, colourless
*V* = 1722.6 (4) Å^3^	0.25 × 0.23 × 0.23 mm
*Z* = 4	

### Data collection

**Table:**

Bruker SMART CCD area-detector diffractometer	3732 independent reflections
Radiation source: fine-focus sealed tube	2017 reflections with *I* > 2σ(*I*)
graphite	*R*_int_ = 0.043
ω scans	θ_max_ = 27.0°, θ_min_ = 2.1°
Absorption correction: multi-scan (*SADABS*; Bruker, 2001)	*h* = −10→10
*T*_min_ = 0.975, *T*_max_ = 0.977	*k* = −20→22
10465 measured reflections	*l* = −15→7

### Refinement

**Table:**

Refinement on *F*^2^	Primary atom site location: structure-invariant direct methods
Least-squares matrix: full	Secondary atom site location: difference Fourier map
*R*[*F*^2^ > 2σ(*F*^2^)] = 0.050	Hydrogen site location: inferred from neighbouring sites
*wR*(*F*^2^) = 0.129	H atoms treated by a mixture of independent and constrained refinement
*S* = 1.02	*w* = 1/[σ^2^(*F*_o_^2^) + (0.0522*P*)^2^ + 0.0211*P*] where *P* = (*F*_o_^2^ + 2*F*_c_^2^)/3
3732 reflections	(Δ/σ)_max_ = 0.001
235 parameters	Δρ_max_ = 0.15 e Å^−^^3^
1 restraint	Δρ_min_ = −0.19 e Å^−^^3^

### Special details

**Table:**

Geometry. All e.s.d.'s (except the e.s.d. in the dihedral angle between two l.s. planes) are estimated using the full covariance matrix. The cell e.s.d.'s are taken into account individually in the estimation of e.s.d.'s in distances, angles and torsion angles; correlations between e.s.d.'s in cell parameters are only used when they are defined by crystal symmetry. An approximate (isotropic) treatment of cell e.s.d.'s is used for estimating e.s.d.'s involving l.s. planes.
Refinement. Refinement of *F*^2^ against ALL reflections. The weighted *R*-factor *wR* and goodness of fit *S* are based on *F*^2^, conventional *R*-factors *R* are based on *F*, with *F* set to zero for negative *F*^2^. The threshold expression of *F*^2^ > σ(*F*^2^) is used only for calculating *R*-factors(gt) *etc*. and is not relevant to the choice of reflections for refinement. *R*-factors based on *F*^2^ are statistically about twice as large as those based on *F*, and *R*- factors based on ALL data will be even larger.

### Fractional atomic coordinates and isotropic or equivalent isotropic displacement parameters (Å^2^)

**Table:**

	*x*	*y*	*z*	*U*_iso_*/*U*_eq_	
N1	0.9655 (2)	0.08862 (10)	0.61461 (15)	0.0408 (4)	
N2	0.8536 (2)	0.03538 (10)	0.56016 (14)	0.0414 (4)	
O1	1.2219 (2)	0.05972 (9)	0.95309 (12)	0.0623 (5)	
H1	1.1561	0.0316	0.9118	0.093*	
O2	1.52034 (18)	0.28449 (9)	0.94432 (11)	0.0499 (4)	
H2	1.5442	0.3149	0.8984	0.075*	
O3	1.01873 (19)	0.01674 (9)	0.77490 (12)	0.0544 (4)	
O4	0.42243 (18)	−0.17176 (9)	0.40757 (13)	0.0562 (4)	
O5	0.29676 (18)	−0.13286 (8)	0.19882 (13)	0.0582 (5)	
O6	0.6296 (2)	0.37824 (9)	0.81028 (13)	0.0639 (5)	
H6	0.5949	0.4222	0.8007	0.096*	
C1	1.1618 (2)	0.13446 (11)	0.77945 (16)	0.0379 (5)	
C2	1.2479 (3)	0.12222 (12)	0.89285 (17)	0.0419 (5)	
C3	1.3640 (3)	0.17364 (13)	0.94638 (17)	0.0445 (5)	
H3	1.4178	0.1655	1.0223	0.053*	
C4	1.4008 (2)	0.23680 (12)	0.88820 (17)	0.0393 (5)	
C5	1.3173 (3)	0.25089 (12)	0.77654 (17)	0.0461 (6)	
H5	1.3401	0.2942	0.7375	0.055*	
C6	1.2005 (2)	0.20011 (12)	0.72414 (17)	0.0442 (6)	
H6A	1.1450	0.2098	0.6490	0.053*	
C7	1.0444 (2)	0.07663 (12)	0.72359 (17)	0.0401 (5)	
C8	0.7848 (2)	0.05124 (12)	0.45725 (18)	0.0412 (5)	
H8	0.8152	0.0956	0.4242	0.049*	
C9	0.6598 (2)	0.00287 (11)	0.38858 (17)	0.0373 (5)	
C10	0.6063 (2)	−0.06345 (11)	0.43485 (17)	0.0408 (5)	
H10	0.6532	−0.0781	0.5098	0.049*	
C11	0.4845 (2)	−0.10693 (11)	0.36970 (18)	0.0414 (5)	
C12	0.4150 (2)	−0.08531 (12)	0.25628 (18)	0.0434 (5)	
C13	0.4679 (3)	−0.02028 (12)	0.21043 (18)	0.0479 (6)	
H13	0.4218	−0.0057	0.1353	0.057*	
C14	0.5908 (3)	0.02357 (12)	0.27733 (17)	0.0443 (5)	
H14	0.6267	0.0674	0.2463	0.053*	
C15	0.4975 (3)	−0.19856 (14)	0.5184 (2)	0.0648 (7)	
H15A	0.6088	−0.2100	0.5209	0.097*	
H15B	0.4437	−0.2441	0.5353	0.097*	
H15C	0.4912	−0.1597	0.5743	0.097*	
C16	0.2220 (3)	−0.11211 (15)	0.0836 (2)	0.0661 (7)	
H16A	0.1795	−0.0610	0.0826	0.099*	
H16B	0.1358	−0.1473	0.0539	0.099*	
H16C	0.3004	−0.1141	0.0369	0.099*	
C17	0.6766 (4)	0.35311 (16)	0.7103 (2)	0.0814 (9)	
H17A	0.7812	0.3736	0.7093	0.122*	
H17B	0.5995	0.3705	0.6436	0.122*	
H17C	0.6812	0.2981	0.7099	0.122*	
H1A	0.979 (2)	0.1297 (8)	0.5719 (15)	0.050*	

### Atomic displacement parameters (Å^2^)

**Table:**

	*U*^11^	*U*^22^	*U*^33^	*U*^12^	*U*^13^	*U*^23^
N1	0.0420 (10)	0.0382 (11)	0.0393 (11)	−0.0067 (9)	0.0028 (8)	−0.0003 (8)
N2	0.0418 (10)	0.0372 (11)	0.0433 (11)	−0.0048 (8)	0.0056 (8)	−0.0044 (8)
O1	0.0787 (13)	0.0582 (11)	0.0429 (10)	−0.0152 (9)	−0.0014 (8)	0.0154 (8)
O2	0.0562 (10)	0.0511 (10)	0.0370 (9)	−0.0089 (8)	−0.0011 (7)	−0.0071 (7)
O3	0.0647 (11)	0.0474 (10)	0.0475 (10)	−0.0106 (8)	0.0049 (8)	0.0089 (7)
O4	0.0589 (10)	0.0405 (9)	0.0654 (11)	−0.0096 (8)	0.0057 (8)	0.0064 (8)
O5	0.0585 (10)	0.0475 (10)	0.0598 (11)	−0.0110 (8)	−0.0056 (8)	−0.0038 (8)
O6	0.0943 (14)	0.0413 (10)	0.0577 (11)	−0.0014 (10)	0.0201 (9)	−0.0022 (8)
C1	0.0427 (12)	0.0373 (12)	0.0311 (12)	0.0030 (10)	0.0031 (9)	0.0000 (9)
C2	0.0522 (13)	0.0417 (13)	0.0321 (12)	0.0043 (11)	0.0099 (10)	0.0038 (10)
C3	0.0528 (13)	0.0486 (14)	0.0286 (12)	0.0043 (11)	0.0012 (10)	−0.0003 (10)
C4	0.0408 (12)	0.0401 (13)	0.0340 (12)	0.0023 (10)	0.0023 (9)	−0.0099 (10)
C5	0.0559 (14)	0.0419 (14)	0.0354 (13)	−0.0025 (11)	−0.0005 (10)	0.0033 (10)
C6	0.0521 (13)	0.0445 (14)	0.0297 (12)	−0.0002 (11)	−0.0043 (10)	0.0016 (10)
C7	0.0419 (12)	0.0399 (13)	0.0367 (13)	0.0026 (10)	0.0048 (10)	0.0000 (10)
C8	0.0415 (12)	0.0352 (12)	0.0453 (14)	−0.0023 (10)	0.0063 (10)	−0.0015 (10)
C9	0.0368 (12)	0.0316 (12)	0.0419 (13)	−0.0005 (9)	0.0053 (9)	−0.0040 (9)
C10	0.0418 (12)	0.0368 (13)	0.0416 (13)	0.0029 (10)	0.0047 (10)	−0.0026 (10)
C11	0.0424 (12)	0.0294 (12)	0.0530 (14)	0.0006 (10)	0.0116 (10)	−0.0015 (10)
C12	0.0414 (12)	0.0339 (12)	0.0513 (15)	−0.0027 (10)	0.0026 (10)	−0.0103 (10)
C13	0.0506 (14)	0.0445 (14)	0.0446 (14)	−0.0028 (11)	0.0022 (11)	−0.0019 (11)
C14	0.0482 (13)	0.0377 (13)	0.0449 (14)	−0.0052 (10)	0.0059 (10)	0.0011 (10)
C15	0.0699 (17)	0.0456 (15)	0.0781 (19)	−0.0015 (13)	0.0147 (14)	0.0184 (13)
C16	0.0569 (15)	0.0674 (18)	0.0628 (18)	−0.0137 (14)	−0.0104 (12)	−0.0078 (13)
C17	0.114 (3)	0.072 (2)	0.0633 (19)	−0.0190 (18)	0.0304 (17)	−0.0166 (15)

### Geometric parameters (Å, °)

**Table:**

N1—C7	1.339 (3)	C5—H5	0.93
N1—N2	1.382 (2)	C6—H6A	0.93
N1—H1A	0.901 (9)	C8—C9	1.456 (3)
N2—C8	1.267 (2)	C8—H8	0.93
O1—C2	1.350 (2)	C9—C14	1.374 (3)
O1—H1	0.82	C9—C10	1.400 (3)
O2—C4	1.367 (2)	C10—C11	1.375 (3)
O2—H2	0.82	C10—H10	0.93
O3—C7	1.254 (2)	C11—C12	1.402 (3)
O4—C11	1.366 (2)	C12—C13	1.377 (3)
O4—C15	1.413 (3)	C13—C14	1.393 (3)
O5—C12	1.363 (2)	C13—H13	0.93
O5—C16	1.426 (3)	C14—H14	0.93
O6—C17	1.410 (3)	C15—H15A	0.96
O6—H6	0.82	C15—H15B	0.96
C1—C6	1.397 (3)	C15—H15C	0.96
C1—C2	1.403 (3)	C16—H16A	0.96
C1—C7	1.469 (3)	C16—H16B	0.96
C2—C3	1.380 (3)	C16—H16C	0.96
C3—C4	1.374 (3)	C17—H17A	0.96
C3—H3	0.93	C17—H17B	0.96
C4—C5	1.384 (3)	C17—H17C	0.96
C5—C6	1.372 (3)		
			
C7—N1—N2	119.58 (17)	C10—C9—C8	121.09 (19)
C7—N1—H1A	125.0 (14)	C11—C10—C9	120.1 (2)
N2—N1—H1A	115.4 (13)	C11—C10—H10	119.9
C8—N2—N1	115.41 (17)	C9—C10—H10	119.9
C2—O1—H1	109.5	O4—C11—C10	124.5 (2)
C4—O2—H2	109.5	O4—C11—C12	115.55 (18)
C11—O4—C15	117.13 (17)	C10—C11—C12	119.9 (2)
C12—O5—C16	116.74 (18)	O5—C12—C13	124.7 (2)
C17—O6—H6	109.5	O5—C12—C11	115.22 (19)
C6—C1—C2	116.94 (18)	C13—C12—C11	120.03 (19)
C6—C1—C7	123.76 (18)	C12—C13—C14	119.6 (2)
C2—C1—C7	119.21 (18)	C12—C13—H13	120.2
O1—C2—C3	117.58 (19)	C14—C13—H13	120.2
O1—C2—C1	121.59 (19)	C9—C14—C13	120.9 (2)
C3—C2—C1	120.83 (19)	C9—C14—H14	119.6
C4—C3—C2	120.42 (19)	C13—C14—H14	119.6
C4—C3—H3	119.8	O4—C15—H15A	109.5
C2—C3—H3	119.8	O4—C15—H15B	109.5
O2—C4—C3	117.89 (18)	H15A—C15—H15B	109.5
O2—C4—C5	121.9 (2)	O4—C15—H15C	109.5
C3—C4—C5	120.2 (2)	H15A—C15—H15C	109.5
C6—C5—C4	119.1 (2)	H15B—C15—H15C	109.5
C6—C5—H5	120.4	O5—C16—H16A	109.5
C4—C5—H5	120.4	O5—C16—H16B	109.5
C5—C6—C1	122.43 (19)	H16A—C16—H16B	109.5
C5—C6—H6A	118.8	O5—C16—H16C	109.5
C1—C6—H6A	118.8	H16A—C16—H16C	109.5
O3—C7—N1	120.01 (19)	H16B—C16—H16C	109.5
O3—C7—C1	121.63 (19)	O6—C17—H17A	109.5
N1—C7—C1	118.35 (19)	O6—C17—H17B	109.5
N2—C8—C9	122.7 (2)	H17A—C17—H17B	109.5
N2—C8—H8	118.7	O6—C17—H17C	109.5
C9—C8—H8	118.7	H17A—C17—H17C	109.5
C14—C9—C10	119.45 (18)	H17B—C17—H17C	109.5
C14—C9—C8	119.44 (19)		

### Hydrogen-bond geometry (Å, °)

**Table:**

*D*—H···*A*	*D*—H	H···*A*	*D*···*A*	*D*—H···*A*
N1—H1A···O2^i^	0.90 (1)	2.22 (1)	3.108 (2)	170 (2)
O6—H6···N2^ii^	0.82	2.55	3.133 (2)	129
O6—H6···O3^ii^	0.82	2.02	2.807 (2)	161
O2—H2···O6^iii^	0.82	1.79	2.599 (2)	171
O1—H1···O3	0.82	1.80	2.534 (2)	148

Symmetry codes: (i) *x*−1/2, −*y*+1/2, *z*−1/2; (ii) −*x*+3/2, *y*+1/2, −*z*+3/2; (iii) *x*+1, *y*, *z*.
